# Draft Genome Sequence of *Lactobacillus reuteri* 121, a Source of α-Glucan and β-Fructan Exopolysaccharides

**DOI:** 10.1128/genomeA.01691-16

**Published:** 2017-03-09

**Authors:** Joana Gangoiti, Xiangfeng Meng, Alicia Lammerts van Bueren, Lubbert Dijkhuizen

**Affiliations:** Microbial Physiology, Groningen Biomolecular Sciences and Biotechnology Institute (GBB), Groningen, The Netherlands

## Abstract

The probiotic bacterium* Lactobacillus reuteri* 121 is a well-known producer of diverse homoexopolysaccharides (α-glucans and β-fructans) from sucrose and maltodextrins/starches of interest for food applications. Here, we report the draft genome sequence of this strain, with a focus on carbohydrate-active enzymes.

## GENOME ANNOUNCEMENT

The exopolysaccharides (EPS) produced by lactic acid bacteria (LAB) are of interest for food applications (1–3). In a previous study, a collection of 182 LAB were screened for the ability to produce EPS in high-sucrose medium (4), resulting in the identification of *Lactobacillus reuteri* 121 as a producer of β-fructans (inulin and levan) and α-glucan (reuteran) EPS. We also have characterized the inulosucrase, levansucrase, and glucansucrase (GS) enzymes converting sucrose into these three different types of EPS (5, 6). *L. reuteri* 121 was also found to encode a GS-like enzyme (designated GtfB) that is inactive on sucrose but displays 4,6-α-glucanotransferase activity (4,6-α-GTase), converting maltodextrins/starch substrates into isomalto-malto polysaccharides (IMMP) (7, 8). Together with this ability to synthesize diverse homo-EPS, *L. reuteri* 121 possesses the generally recognized as safe status, opening great possibilities for its application in the food industry.

Here, we present the draft genome sequence of *L. reuteri* 121, which was obtained from an 8- to 12-kb insert library constructed and sequenced using a PacBio RS II instrument at GATC Biotech AG (Konstanz, Germany). A total of 55,989 reads with a mean size of 5,482 bp were obtained, providing 105,77-fold genome coverage. *De novo* assembly was performed by PacBio SMRT Analysis 2.0 using the HGAP2 protocol (Pacific Biosciences, USA), yielding 14 contigs. The largest contig was 1,570,268 bp long. The genome sequence was annotated using the NCBI Prokaryotic Genome Annotation Pipeline (PGAP) (https://www.ncbi.nlm.nih.gov/genome/annotation_prok/) (9) and the Rapid Annotations using Subsystems Technology (RAST) server (http://rast.nmpdr.org/) (10). The draft genome of *L. reuteri* 121 is 2,302,234 bp in length and has an average G+C content of 39.0%, similar to that of other *L. reuteri* sequenced genomes (11, 12). A total of 2,226 genes (2,027 protein-coding sequences, 105 pseudogenes, and 94 RNA-encoding genes) were annotated using the NCBI annotation pipeline. Because the majority of enzymes involved in EPS synthesis in LAB fall within the carbohydrate active enzyme (CAZy) classification, we analyzed the *L. reuteri* 121 genome by dbCAN (http://csbl.bmb.uga.edu/dbCAN/) (13), which resulted in the identification of 26, 25, and 12 putative glycoside hydrolases (GH), glycosyl transferases (GT), and carbohydrate esterases, respectively. Consistent with previous studies, the genes of two GH68 proteins (levansucrase and inulosucrase) and two GH70 proteins (4,6-α-GTase and GS) were identified. However, the *L. reuteri* 121 genome does not appear to encode many (extracellular) enzymes involved in the degradation of β-fructans or α-glucans, and only a single GH31 enzyme was predicted to function as an extracellular α-glucosidase. In contrast, two extracellular β-xylosidases and an extracellular α-*N*-arabinofuranosidase were found belonging to the GH120 and GH43 families, respectively. These enzymes may be involved in the degradation of arabinose- and xylose-containing polysaccharides and/or oligosaccharides, which are recognized as promising prebiotics present in plant cell walls (14–16). Furthermore, genome analysis using antiSMASH 3.0 (17, 18) revealed two heteropolysaccharide biosynthesis gene clusters, both containing several GT enzymes. This finding indicates that *L. reuteri* 121 holds a great potential for the production of both homo- and heteropolysaccharides.

### Accession number(s).

This whole-genome shotgun project has been deposited at DDBJ/ENA/GenBank under the accession no. MKQH00000000. The version described in this paper is version MKQH01000000.
